# Plattformtechnologien in der Impfstoffentwicklung

**DOI:** 10.1007/s00103-025-04024-6

**Published:** 2025-03-04

**Authors:** Eberhard Hildt

**Affiliations:** Paul-Ehrlich-Institut, Paul-Ehrlich-Str. 51–59, 63225 Langen (Hessen), Deutschland

**Keywords:** Impfstoff, Plattformtechnologie, Virale Vektoren, mRNA, Nanopartikel, Vaccine, Platform technology, Viral vectors, mRNA, Nanoparticles

## Abstract

Als Plattformtechnologien im engeren Sinn werden nachfolgend Ansätze der Impfstoffentwicklung bezeichnet, bei denen der Impfstoff stets auf einem identischen Grundgerüst basiert und sich nur hinsichtlich des Antigens unterscheidet. Ein Vorteil von Plattformtechnologien besteht in der raschen Anpassbarkeit dieser Technologien für die Entwicklung eines Impfstoffs gegen neuartige Erreger oder Varianten. Bei derzeit in der EU zugelassenen Impfstoffen werden virale Vektoren und mRNA als Plattformen verwendet. Als virale Vektoren dienen dabei rekombinante Adenoviren (Ad), das vesikuläre Stomatitis-Virus (VSV) und das modifizierte Vacciniavirus Ankara (MVA). Die Applikation von mRNA-basierten Impfstoffen erfolgt in Form von Lipidnanopartikeln (LNPs). Die Funktion der LNPs besteht in dem Schutz der mRNA vor Abbau, der Förderung der Aufnahme der mRNA in die Zellen und einer Adjuvanswirkung.

## Abstract (en)

Platform technologies in the narrower sense refer to approaches to vaccine development in which the vaccine is always based on a consistently identical framework and differs only in terms of the antigen. One advantage of platform technologies is their rapid adaptability for the development of a vaccine against novel pathogens or variants. Currently approved vaccines in the EU use viral vectors and mRNA as platforms. Recombinant adenoviruses (Ad), vesicular stomatitis virus (VSV), and modified vaccinia virus Ankara (MVA) serve as viral vectors. The application of mRNA-based vaccines is carried out in the form of lipid nanoparticles (LNPs). The function of the LNPs is to protect the mRNA from degradation, promote the uptake of the mRNA into the cells, and provide an adjuvant effect.

## Einleitung

Eine entscheidende Herausforderung beim Auftreten neuartiger pandemischer Erreger ist die Entwicklung sicherer und wirksamer Impfstoffe, um die Verbreitung des Erregers zu verlangsamen bzw. im günstigsten Fall zu stoppen. Neben der Entwicklung, Charakterisierung und Überprüfung der Wirksamkeit und Sicherheit von Impfstoffen in klinischen Studien ist der Zulassungsprozess ein wesentlicher Zeitfaktor. Hierbei kann durch Modifizierungen im Zulassungsverfahren, wie bspw. ein „Rolling-Review“^1^, das Verfahren verkürzt werden und ggf. eine bedingte Zulassung erfolgen. Dabei werden die Grundprinzipien des Zulassungsprozesses zur Überprüfung der Sicherheit und Wirksamkeit nicht aufgeweicht.

Plattformtechnologien (Infobox 1) kommt für eine angestrebte schnelle Verfügbarkeit eines Impfstoffs vor dem Hintergrund einer pandemischen Situation eine besondere Bedeutung zu. Als Plattformtechnologie werden nachfolgend Ansätze bezeichnet, die auf einem stets identischen Grundgerüst basieren und sich nur hinsichtlich des Antigens unterscheiden. Dies können Vektorimpfstoffe sein, die auf modifizierten Viren basieren [1], wie auch mRNA- oder DNA-basierte Impfstoffe und Nanopartikel als Antigenträger, die flexibel mit dem jeweils relevanten Antigen beladen werden können.

Die Ebola- und Zika-Virus-Epidemie und insbesondere die SARS-CoV-2-Pandemie waren wesentliche Faktoren, die zur Entwicklung und Etablierung dieser Technologien beigetragen haben.

Der vorliegende Übersichtsartikel fokussiert sich in erster Linie auf Plattformtechnologien, die bereits Anwendung bei der Entwicklung viraler Impfstoffe gefunden haben. Hierbei werden knapp die Grundlagen der jeweiligen Technologie und Anwendungsbeispiele beschrieben. Die vorgestellten Technologien umfassen virale Plattformen auf der Basis rekombinanter Viren: Adenoviren (Ad), vesikuläres Stomatitis-Virus (VSV) und modifiziertes Vacciniavirus Ankara (MVA). Die viralen Plattformen auf der Basis rekombinanter Adenoviren werden am Beispiel von Impfstoffen gegen das Ebolavirus und SARS-CoV‑2 beschrieben. Weiterhin werden der MVA-Impfstoff gegen das Ebolavirus und das vesikuläre Stomatitis-Virus (VSV) als Impfstoffplattform gegen das Ebolavirus vorgestellt. Auf mRNA-basierte Impfstoffe wird am Beispiel der SARS-CoV-2-Impfstoffe und eines Impfstoffs gegen das respiratorische Synzytial-Virus (RSV) eingegangen und bei der Beschreibung DNA-basierter Impfstoffe wird ein Impfstoff gegen SARS-CoV‑2 beschrieben. Abschließend werden neuartige Ansätze, die auf verschiedenen Nanopartikeln basieren und sich in unterschiedlichen Phasen der Entwicklung befinden, hinsichtlich ihrer Technologie und ihres möglichen Potenzials, als Impfstoffplattform zu fungieren, beschrieben.

Einen Überblick über Funktionsweisen, Anwendungsbeispiele und Vorteile der 4 beschriebenen Plattformtechnologien bietet Tab. 1.

**Tab. 1:** Plattformtechnologien in der Impfstoffentwicklung

Funktionsweise	Vorteile	Beispiele
*Virale Vektorimpfstoffe*		
Basieren auf der Verwendung von gentechnisch modifizierten Trägerviren (Vektoren), die nicht (mehr) pathogen sind	Schnelle Entwicklung und Anpassung an neue Erreger	Adenovirale SARS-CoV-2-Impfstoffe und Ebola-Impfstoffe
Genetisches Material (RNA oder DNA), das für Antigen eines anderen Erregers codiert, wird in menschliche Zellen eingeschleust	Effiziente Immunantwort durch natürliche Viruseigenschaften	VSV- und MVA-basierte Ebola-Impfstoffe
Ausgenutzt wird die Fähigkeit dieser modifizierten Viren, in Zellen einzudringen und temporär genetische Information zu übertragen, sodass diese Antigene gebildet werden, ohne dabei eine Krankheit auszulösen	Korrekte Faltung und Glykosylierung viraler Hüllproteine	Gelbfiebervirus-basierte Impfstoffe gegen Dengue-Fieber
Dabei wird sichergestellt, dass das gentechnisch veränderte Virus nicht mehr oder nur zeitlich begrenzt replikationsfähig ist		
Das Immunsystem erkennt dies/e Antigen/e, bildet Antikörper dagegen und löst die zelluläre Immunantwort aus (T-Zellen)		
Als Vektoren dienen u. a. Adenoviren (Ad), vesikuläres Stomatitis-Virus (VSV), modifiziertes Vacciniavirus Ankara (MVA)		
*mRNA-Impfstoffe*		
Basieren auf der Verwendung von Boten-Ribonukleinsäure (mRNA)	Einfache Anpassung an neue Erregervarianten	SARS-CoV-2-Impfstoffe
mRNA-Impfstoffe codieren für ein spezifisches Protein/Antigen des Erregers in Form von mRNA	Beschleunigte Entwicklung und Produktion	RSV-Impfstoff
Diese mRNA wird in Lipidnanopartikel (LNP) verpackt, um sie vor Abbau zu schützen und in das Zielgewebe einzuschleusen	Kostengünstige Herstellung	
Nach der Aufnahme in die Zelle wird die mRNA in das von ihr codierte Antigen übersetzt (translatiert) und somit dem Immunsystem zum Auslösen einer Immunantwort präsentiert (humorale und zelluläre Immunantwort)	Intrinsische Adjuvanswirkung durch die mRNA und LNP-Komponenten	
Dabei üben die mRNA selbst und LNP-Komponenten eine Adjuvanswirkung aus	Korrekte Faltung und Glykosylierung der rekombinanten Antigene	
*DNA-Impfstoffe*		
Basieren auf der Verwendung von Desoxyribonukleinsäure (DNA)	Einfache Anpassung an neue Erregervarianten	SARS-CoV-2-Impfstoff
DNA-Impfstoffe codieren für ein spezifisches Protein/Antigen des Erregers in Form von DNA. Die genetische Information liegt in Form eines zirkulären DNA-Moleküls (Plasmid oder Minicircle-DNA) vor	Beschleunigte Entwicklung und Produktion	
Diese DNA wird entweder direkt in Muskelgewebe oder subkutan injiziert oder in Lipidnanopartikel (LNP) verpackt oder mechanisch (hoher Druck oder an Goldpartikel absorbiert) in das Zielgewebe eingeschleust	Kostengünstige Herstellung	
Nach der Aufnahme in die Zelle wird die DNA in mRNA übersetzt und diese dann in das von ihr codierte Antigen translatiert und somit dem Immunsystem zum Auslösen einer Immunantwort präsentiert (humorale und zelluläre Immunantwort)	Intrinsische Adjuvanswirkung durch die DNA	
Dabei übt die DNA eine intrinsische Adjuvanswirkung aus	Korrekte Faltung und Glykosylierung der rekombinanten Antigene	
*Antigen-beladene Nanopartikel*		
Impfstoffe, die auf Nanopartikeln basieren, nutzen Strukturen im Nanometerbereich als Antigenträger	Einfache Anpassung an neue Erregervarianten	(Impfstoffe gegen Influenza oder Betacoronaviren befinden sich derzeit in der Entwicklung)
Dabei kann es sich um anorganische oder chemisch-synthetisch hergestellte Polymere, Lipidvesikel, selbstaggregierende Proteine oder virale Strukturproteine, die Partikel bilden, sogenannte VLPs (Virus-Like Particles) handeln	Modulares System: ggf. kann das Innere der Nanopartikel mit Adjuvanzien beladen werden	
Die hoch geordnete Struktur der Antigene, die auf der Oberfläche dieser als Antigenträger fungierenden Nanopartikel fixiert sind, bedingt eine robuste Induktion der Immunantwort, insbesondere der B‑Zellantwort (Antikörperantwort)	Potenzial zur Induktion einer breiten Immunantwort	
Dabei ist neben der Beladung mit nur einem Antigen auch die Beladung mit verschiedenen Antigenen möglich	Proteinbasierter Impfstoff, daher ggf. leichtere Akzeptanz in weiteren Teilen der Bevölkerung	
Durch das selektive Erkennen von konservierten Bereichen in den verschiedenen Antigenen durch die B‑Zellrezeptoren kann die Schutzwirkung der ausgelösten Immunantwort verbreitert werden		

## Virale Vektorimpfstoffe

### Rekombinante Adenoviren

Adenoviren sind ca. 80–100 nm große nicht umhüllte Viren. Sie besitzen ein lineares doppelsträngiges DNA-Genom einer Größe von ca. 26–45 kB. Das Viruskapsid wird aus sog. Kapsomeren aufgebaut. Dabei können 2 Typen von Kapsomeren unterschieden werden: Pentone und Hexone. Ein auffälliges Strukturmerkmal von Adenoviren ist das Vorhandensein sog. Fiberproteine, die eine wesentliche Rolle bei der Bindung an die Zielzelle spielen. Als Rezeptoren für Adenoviren fungieren u. a. verschiedene Integrine wie αvβ3, αvβ5 und α5β1 sowie CAR (Coxsackievirus and Adenovirus Receptor; [2]).

Die Fiberproteine sind auch eine wesentliche Zielstruktur für die Bindung neutralisierender Antikörper. Ein Problem bei der Anwendung rekombinanter Adenoviren als Impfstoffplattform ist das Vorhandensein neutralisierender Antikörper in weiten Teilen der Bevölkerung gegen eine Reihe von hochprävalenten Serotypen. Daher werden bei der Anwendung von rekombinanten Adenoviren als Impfstoffplattformen nichthumane Adenoviren wie ein Schimpansenadenovirus oder Serotypen, die nur eine sehr geringe Prävalenz aufweisen, verwendet.

Adenoviren bieten sich aus verschiedenen Gründen als virale Vektoren an. Dazu zählen bspw. der episomale, nicht integrierende Charakter des viralen Genoms, die Möglichkeit der Insertion sehr großer Transgene oder die Möglichkeit, sowohl replikationskompetente als auch replikationsinkompetente Viren zu produzieren. Als Impfstoffplattform dienen derzeit in erster Linie jedoch replikationsinkompetente Viren, bei denen die für die viralen Proteine E1 und E3 codierenden Genombereiche deletiert sind. Durch homologe Rekombination wird der für E1 codierende Bereich durch die für das Impfantigen codierende Sequenz ersetzt. Rekombinante Adenoviren der zweiten Generation weisen auch Deletionen im Bereich E2 und/oder E4 auf, um die Transgenkapazität zu erhöhen. Die Replikation und Herstellung dieser selbst nicht replikationskompetenten rekombinanten Adenoviren erfolgt mittels sog. Verpackungszelllinien, welche die für das virale E1-Protein codierende Sequenz als Transgen tragen. Dabei finden HEK293T-Zellen sowie PERC.6-Zellen Verwendung [3–5].

Adenoviren lösen nach ihrer Applikation am Injektionsort eine Induktion der angeborenen Immunantwort aus und führen so zu einer lokalen inflammatorischen Reaktion. Dies wiederum bedingt die Einwanderung von Immunzellen, die dann das rekombinante Adenovirus aufnehmen. Nach Aufnahme des rekombinanten Adenovirus in die Immunzellen kommt es zur Expression des rekombinanten adenoviralen Genoms und somit zur Expression des Integrats, das für das jeweils interessierende Antigen codiert, sodass es in diesen Zellen zur Expression des Antigens kommt. Die Immunzellen wandern dann in die benachbarten Lymphknoten, wo es zur Induktion einer B‑ und T‑Zellantwort kommt [6].

Zabdeno® erhielt am 01.07.2020 als erster Adenovirus-basierter Impfstoff eine Zulassung der Europäischen Union (EU). Es handelt sich dabei um einen Ad26-basierten Impfstoff gegen das Ebolavirus. Zabdeno® findet Verwendung in einem „Prime-Boost-Impfregime“ basierend auf einer Erstimpfung (Prime) mit Zabdeno® und einer Zweitimpfung (Boost) mit MVA-BN-Filo (also einem MVA-basierten Vektorimpfstoff, siehe unten).

Die Covid-19-Pandemie führte zur Entwicklung weiterer adenoviraler Vektorimpfstoffe. Zu erwähnen sind hier der auf einem Schimpansenadenovirus basierende Impfstoff ChAdOx1 (AZD122, Vaxzevria) sowie der auf dem humanen Serotyp Ad26 basierende Impfstoff Ad26CoV2S (JNJ-78436735, Jcovden). Beide erhielten eine bedingte Zulassung in der EU. Auf Antrag des Herstellers, begründet mit geringen Verkaufszahlen, wurde im Frühjahr 2024 die Zulassung für Vaxzevria zurückgenommen. Der auf Ad5 basierende Impfstoff Ad5nCoV von CanSino sowie der auf Ad26 *(Prime)* und Ad5 *(Boost)* basierende Impfstoff Sputnik V (Gam-COVID-Vac) verfügen über keine EU-Zulassung.

Im Zusammenhang mit der Verimpfung der Covid-19-Impfstoffe „ChAdOx1 nCoV-19“ (Astra-Zeneca) und „Covid-19 Vaccine Janssen“ (Ad26.COV2.S) wurden Ende Februar 2021 verschiedene Thrombosen berichtet, darunter u. a. cerebrale Sinusvenenthrombose (CVST) im Zusammenhang mit Thrombozytopenie. Als ursächlich wurden „platelet-activating antibodies“ gegen die Plättchenzytokine PF4 und CXCL4 identifiziert. Diese Nebenwirkung wurde als VITT („vaccine-induced immune thrombotic thrombocytopenia“) bezeichnet. Sie trat mit einer Häufigkeit von ca. 1 zu 100.000 auf und verlief in 23–40 % aller berichteten Fälle tödlich [7–9].

### Rekombinantes vesikuläres Stomatitis-Virus (VSV)

VSV als Basis eines Vektorimpfstoffs ist eng mit der Entwicklung eines Impfstoffs gegen das Ebolavirus verbunden. Nachdem bereits erfolgreiche „Proof-of-principle“-Experimente zu VSV-basierten Vektorimpfstoffen gegen Filoviren (bspw. Ebolavirus) Anfang des Jahrtausends durchgeführt worden waren, fehlten weitere Phase-II- und Phase-III-Untersuchungen, um die Wirksamkeit dieser Impfstoffe zu belegen. Im Zuge der epidemischen Ebolavirus-Ausbrüche 2013–2016 in Westafrika wurden die entsprechenden Studien durchgeführt, welche die hohe Wirksamkeit dieser Impfstoffe zeigten [10–13].

Darauf basierend konnte im November 2019 eine bedingte Zulassung für den VSV-basierten Impfstoff gegen das Ebolavirus (Ervebo®) für die EU erteilt werden. Im Dezember 2019 folgte die Zulassung durch die U.S. Food and Drug Administration (FDA) für die USA. Die Bedingtheit der Zulassung basiert auf dem angewandten „PRIME“-Verfahren, das es ermöglicht, dass für die Zulassung dringend benötigte Arzneimittel einzelne Informationen zum Herstellungsprozess innerhalb weniger Monate nach Erteilung der bedingten Zulassung nachgereicht werden können.

VSV (Vesicular Stomatitis Virus) gehört zur Gruppe Vesikulovirus und besitzt somit ein nichtsegmentiertes einzelsträngiges RNA-Genom negativer Polarität, das für 5 Strukturproteine codiert. Dabei handelt es sich um die RNA-abhängige RNA-Polymerase (L), das Nukleoprotein (N), das Matrixprotein (M), das Phosphoprotein (P) und das Glykoprotein (G). Das N‑Protein komplexiert das virale Genom. P verknüpft das L‑Protein mit N und baut so das Kapsid auf. M spielt eine wesentliche Rolle für die helikale Verpackung der genomischen RNA. VSV ist ein umhülltes Virus. G fungiert in seiner trimeren Form als Hüllprotein und vermittelt die Bindung an den LDL‑R (Low Density Lipoprotein Receptor), der ubiquitär exprimiert wird, sodass VSV keine ausgeprägte Gewebsspezifität aufweist. Der komplette Replikationszyklus von VSV geht mit der Zerstörung der infizierten Zelle einher.

VSV-Infektionen lösen bei Huftieren vesikuläre Stomatitis aus. Zwar heilt die Infektion häufig aus, allerdings kann es durch die Gewebszerstörung zu Verletzungen im Bereich der Hufe kommen.

VSV eignet sich u. a. aufgrund der geringen Größe seines Genoms von ca. 11 kB als Vektor. Dabei sind Insertionen fremder Sequenzen bis zu einer Größe von 5 kB möglich. Die Entwicklung eines Plasmid-basierten reversen Genetiksystems zur leichten und effizienten Manipulation des VSV-Genoms ist ein weiterer wesentlicher Faktor, der dafür spricht, VSV als Vektor zu benutzen, wie auch die nahezu fehlende Pathogenität von VSV bei Infektionen von Menschen. Schließlich ist VSV in großen Mengen in Zellkultur produzierbar [14].

Grundsätzlich können sowohl replikationskompetente als auch replikationsinkompetente VSV-Derivate als Impfstoff Verwendung finden. Im Falle replikationskompetenter VSV-Plattformen ist jedoch zu bedenken, dass die geimpften Personen für einige Zeit das Virus ausscheiden und somit ein Risiko für Nutztierbestände darstellen können [15].

Die Verwendung von replikationskompetenten als auch replikationsinkompetenten VSV als Impfstoffplattform basiert auf dem Verlust von G. Bei replikationsinkompetenten Viren erfolgt die Bildung des Antigens, das G ersetzt durch eine Verpackungszelllinie in trans, sodass pseudotypisierte G‑defiziente VSV-Rekombinanten entstehen, die statt G das gewünschte Antigen (Oberflächenprotein eines anderen Virus) als Hüllprotein tragen [1, 15].

Bei replikationskompetenten VSV-Rekombinanten ist die für G codierende Sequenz durch die für das Antigen codierende Sequenz ersetzt. Auch hier entstehen pseudotypisierte VSV-Partikel. Durch das Immunsystem wird die Replikation gehemmt, sodass nach wenigen Replikationsrunden das rekombinante Virus eliminiert ist.

### Modifiziertes Vacciniavirus Ankara (MVA)

Bei MVA handelt es sich um ein attenuiertes Vacciniavirus, das zur Familie der Pockenviren gehört. Im Zuge der seriellen Passage des Ausgangsvirus „Chorioallantois Vaccinia Virus Ankara“ (CVA) konnte nach 371 Passagen eine verminderte Virulenz beobachtet werden. Nach Passage 516 wurde das vielfältig mutierte Virus dann als „Modified Vaccinia Virus Ankara“ (MVA) bezeichnet. Eine Infektion des Menschen mit MVA verursacht keine Pockennarben. MVA ist in nahezu allen Säugerzellen nicht mehr replikationskompetent, kann aber in avianen Zellen vermehrt werden. Die fehlende Replikationsfähigkeit und Verlust der Pathogenität, u. a. bedingt durch vielfältige Deletionen im viralen Genom, was zu einer Verminderung der Genomgröße von 204,5 kb auf 178 kb führt, sowie das Vorhandensein von etablierten Werkzeugen zur Manipulation des viralen Genoms machen MVA zu einem geeigneten Vektor, in den die für das interessierende Antigen codierende Sequenz integriert werden kann. Da durch die Deletionen eine Reihe von viralen Genen betroffen sind, welche die Evasion vor der angeborenen Immunität vermitteln und die MVA-Infektion zur Induktion von Apoptose führt, ist die Expression des Transgens zeitlich begrenzt, da die Eliminierung des Vektors durch die Immunmechanismen des Wirts erfolgen kann. Umgekehrt übt die so mögliche Aktivierung der angeborenen Immunität einen Adjuvanseffekt aus [16–19].

Die Herstellung rekombinanter MVA-Viren kann entweder mittels homologer Rekombination zwischen einem Transfervektor und der viralen DNA erfolgen oder auf der Basis von klonierten MVA-Genomen in Form von BACs (Bacterial Artificial Chromosomes) in *E. coli* durchgeführt werden.

MVA dient als Impfstoffplattform für eine Impfstoffkomponente gegen das Ebolavirus. Die Impfung basiert auf einem heterologen Prime-Boost-Ansatz. Dabei erfolgt die Erstimpfung mit einem adenoviralen Vektor (Ad26.ZEBOV, Zabdeno®), gefolgt von einer Zweitimpfung mit MVA-BN-Filo (Mvabea®). Die Zulassung seitens der Europäischen Arzneimittel-Agentur (EMA) erfolgte 2020 [20–22].

## mRNA-basierte Impfstoffe

Der Ansatz, mRNA (Boten-Ribonukleinsäure) als Grundlage für therapeutische und präventive Impfstoffe zu verwenden, ist nicht neu, jedoch haben die Entwicklung und Zulassung mRNA-basierter Impfstoffe durch die SARS-CoV-2-Pandemie einen wesentlichen Schub bekommen.

So konnte die Entwicklung mRNA-basierter Impfstoffe zu diesem Zeitpunkt auf Arbeiten zu mRNA-basierten Impfstoffen bspw. gegen Rabies, Zika-Virus oder Influenza aufbauen. Im Rahmen erster klinischer Studien konnten bereits wesentliche Erkenntnisse zur Herstellung und zum Sicherheitsprofil (Verträglichkeit, Dosisfindung) dieser Impfstoffe gewonnen werden. Weiterhin war bereits eine Reihe therapeutischer mRNA-Impfstoffe gegen verschiedene Tumoren entwickelt und teilweise auch bereits in klinischen Studien der Phase I und II erprobt worden [23–26].

Stand Mitte des Jahres 2024 befinden sich über 150 mRNA-basierte Impfstoffe gegen unterschiedliche virale Erreger in verschiedenen Phasen der klinischen Prüfung (Phase I bis III). Derzeit verfügen 9 mRNA-basierte Virusimpfstoffe über eine Zulassung. Dabei handelt es sich zum größten Teil um Covid-spezifische Impfstoffe, aber mit dem mRNA-basierten Impfstoff mRNA-1345 (mResvia®) gegen RSV von Moderna erhielt der erste nicht-Covid-spezifische mRNA-Impfstoff im Sommer 2024 eine Zulassungsempfehlung durch die EMA. Dieser RSV-Impfstoff (mRNA-1345) basiert auf einer mRNA, die für das in der Präfusionskonformation stabilisierte Glykoprotein F(preF) des RSV codiert. mRNA-1345 verfügt über eine Zulassung zur Immunisierung von Personen ab 60 Jahren [27, 28].

Ende 2020/Anfang 2021 wurden die ersten beiden mRNA-basierten Impfstoffe „bnt162b2“ (BioNTech/Pfizer) und „mRNA-1273“ im Rahmen einer Notfallzulassung (Emergency Use Authorization, EUA) in den USA und auf der Basis einer bedingten Zulassung (Conditional Approval) für die EU zugelassen [24, 25, 29, 30].

Die aktuell relevanten mRNA-basierten Impfstoffe bestehen aus 2 Komponenten: der mRNA und dem Verpackungssystem, das auf Lipiden basiert und die Grundlage für die Bildung mRNA-beladener Lipidnanopartikel (LNPs) darstellt. Die Herstellung der mRNA erfolgt derzeit durch *In-vitro*-Transkription mittels T7-Polymerase. mRNA-Impfstoffe verfügen zur Erhöhung der Translationseffizienz und zu ihrer Stabilisierung über ein 5’Cap (Aufsatz am 5’-Ende), ein 5’UTR (5’ untranslatierte Region) sowie über einen Poly(A)-Schwanz am 3’-Ende. Bei der Herstellung von mRNAs werden entweder unmodifizierte oder modifizierte Nucleotide verwendet, Letztere um die Reaktogenität (Fähigkeit, Reaktionen hervorzurufen) zu vermindern. mRNA verfügt per se über eine gewisse Reaktogenität, bedingt durch die Induktion der angeborenen Immunität. Die Verwendung modifizierter Basen und die Reduktion des Anteils von doppelsträngiger RNA (dsRNA) können zur Verminderung der Reaktogenität führen. Die Reinigung des Endprodukts zum Abtrennen von Syntheseabbruchprodukten, DNA-Resten und Nucleotiden ist ein wesentlicher Faktor zur Verminderung der Reaktogenität. Eine chemische Totalsynthese hinreichender Mengen an mRNA für Impfkampagnen ist derzeit noch nicht etabliert [31, 32].

Bei mRNA handelt es sich um ein vergleichsweise instabiles Molekül, das durch verschiedene Enzyme leicht abgebaut werden kann. Daher erfüllt die Verpackung der mRNA in LNPs verschiedene Funktionen: Zum einen dient sie dem Schutz vor der Aktivität RNA-abbauender Enzyme, aber zum anderen auch dem Transfer der verpackten mRNA über die Plasmamembran in die Zelle. Mithin wirken die LNPs als „Transfektionsreagenz“. LNPs bestehen in der Regel aus Cholesterol, verschiedenen kationischen Lipiden und Polyethylenglycol-(PEG-)konjugierten Lipiden [31]. Neben den beiden oben beschriebenen Funktionen üben diese Komponenten aber teilweise, wie insbesondere auch die RNA selbst, eine deutliche Adjuvanswirkung aus. Sämtliche derzeit zugelassenen mRNA-Impfstoffe sind unadjuvantiert. Dies ist u. a. aufgrund der den Lipidkomponenten und insbesondere der mRNA innewohnenden Adjuvanswirkung möglich. Die Adjuvanswirkung der mRNA basiert stark auf der Stimulation der angeborenen Immunität, indem sog. PRRs (Pattern Recognition Receptors) aktiviert werden. Dabei erkennen die PRRs Toll-Like Receptor(TLR)3, 7 und 9, sowohl dsRNA als auch ssRNA, während TLR8 ausschließlich ssRNA erkennt. Darüber hinaus fungieren MDA‑5, LGP2 und RIG‑I als RNA-Sensoren [33]. Wie bereits erwähnt sind das Vermeiden von dsRNA-Strukturen sowie die Verwendung von modifizierten Nucleotiden ein Weg, um eine zu starke Reaktogenität zu vermeiden. Anstelle von Uracil wird häufig N1-Methlypseudouridin verwendet. Werden nichtmodifizierte Nucleotide verwendet, so erlaubt dies in der Regel nur die Verwendung geringerer mRNA-Mengen im Vergleich zu mRNAs basierend auf modifizierten Nucleotiden [34, 35].

Im Unterschied zu DNA-basierten Impfstoffen ist kein Transfer der mRNA in den Zellkern erforderlich; die mRNA wird im Zytosol translatiert. Ebenso erfolgen keine posttranskriptionellen Prozesse mehr, da Spleißen keine Rolle spielt. Obgleich in eukaryontischen Zellen keine Aktivität der reversen Transkriptase bspw. in Form von Line‑1 vorhanden ist, gibt es bis dato keine Hinweise auf eine Integration von so entstandenen DNAs in das Wirtsgenom, weder nach Impfung noch nach durchgemachter Infektion [36].

Zwar können mRNAs leicht in größeren Mengen hergestellt werden, dennoch kann es aus unterschiedlichen Gründen sinnvoll sein, mit verminderten RNA-Mengen zu arbeiten. So kann bspw. unter den Bedingungen einer Pandemie die Verfügbarkeit sowohl der zulassungskonformen Reagenzien für die Bildung von LNPs als auch der Materialien für die *In-vitro*-Transkription begrenzt sein.

Sich selbst amplifizierende „saRNAs“ stellen einen Ansatz dar, diesen Aspekt zu adressieren. Dazu werden hauptsächlich 2 Ansätze verfolgt. Die Amplifizierung der für das Antigen codierenden mRNA erfolgt mittels eines alpha-viralen Replikons. Die für das Amplicon codierende Sequenz kann entweder direkt auf derselben RNA wie die für das Antigen codierende Sequenz liegen oder auf einer separaten mRNA, die dann co-transfiziert wird. In diesem Fall spricht man von „trans amplifying“ mRNA (taRNA). Neben dem Vorteil der Mengenreduktion erlaubt die Verwendung sich selbst amplifizierender RNA die längere Expression des Antigens. Dennoch kommt es mit der Zeit bedingt durch Mechanismen der angeborenen Immunität („innate immunity“) zum Verlust der Replikationsmaschinerie und der für das Antigen codierenden mRNA [37–39].

mRNA-basierte Impfstoffe lösen gleichermaßen eine humorale (Antikörper) wie auch eine T‑Zellantwort aus, basierend auf der Induktion von CD8+-T-Zellen, da das Antigen aufgrund der LNP-vermittelten Transfektion auch direkt in antigenpräsentierenden Zellen (APCs) produziert und präsentiert wird [33].

Ein weiterer Vorteil der mRNA-basierten Plattform ist ihre leichte Adaptierbarkeit an neue Varianten, was insbesondere bei Erregern mit hoher genetischer Variabilität wie SARS-CoV‑2 oder Influenza von großer Bedeutung ist. Der Plattformcharakter dieser Technologie erlaubt eine leichtere Anpassung des Impfstoffs an den Erreger, da nur die codierende Sequenz zu verändern ist [40, 41].

Aufgrund der bisher noch relativ kurzen Anwendungsdauer von mRNA-basierten Impfstoffen ist noch nicht vollkommen klar, ob sich mRNA-basierte Impfstoffe hinsichtlich der Gedächtnisfunktion des Immunsystems von „konventionellen“ Impfstoffen wie Inaktivatvirus oder proteinbasierten Impfstoffen unterscheiden. Es zeigte sich, dass nach mRNA-basierter Impfung gegen Covid-19 in der Fraktion der langlebigen Plasmazellen (LLPCs) nur sehr wenige antikörpersezernierende Zellen (ASCs) nachweisbar waren. Allerdings ist noch nicht abschließend geklärt, ob dies ein genereller Effekt ist, der für das Spike-Protein von SARS-CoV‑2 spezifisch ist, also auch nach Infektion so auftritt, oder ob es ein spezifisches Problem der mRNA-basierten Impfung gegen Covid-19 ist [42].

Derzeit ist die Verwendung mRNA-basierter Impfstoffe noch an eine komplexere Infrastruktur gekoppelt, bedingt durch die vergleichsweise geringe Stabilität, die eine längerfristige Lagerung bei −80 °C (bnt162b) oder −20 °C (mRNA1273) erfordert.

Ein interessanter möglicher Aspekt bei der Verwendung von mRNA-Impfstoffen ist die Verbreiterung der Immunantwort bspw. gegen verschiedene Influenza-Subtypen. Dies kann durch multivalente mRNA-Impfstoffe erfolgen, also Impfstoffe, in denen mRNAs, die für das Hämagglutinin (HA) verschiedener Influenza-Subtypen codieren, gemischt werden [43]. Aufgrund der geringen RNA-Mengen, die für eine robuste Induktion einer Immunantwort erforderlich sind, ist dies leichter möglich, ohne dass durch eine zu große Gesamt-RNA-Menge Nebenwirkungen ausgelöst werden, als im Falle Protein-basierter (Spalt‑)Impfstoffe. Bei den Protein-basierten Impfstoffen würde in diesem Fall die tolerierbare Antigenmenge überschritten werden.

## DNA-Impfstoffe

Bereits nach der intramuskulären Injektion unkomplexierter („nackter“) DNA in Muskeln kann die Bildung von Antikörpern gegen das von der DNA codierte Antigen beobachtet werden. Die Applikationsart hat jedoch naturgemäß einen entscheidenden Einfluss auf die Expressionsstärke des Antigens. So kommt es nach subkutaner Injektion von DNA-Impfstoffen zur Aufnahme der DNA in Langerhans-Zellen und dendritischen Zellen, die dann zu den lokalen Lymphknoten wandern. Dort wird das exprimierte Antigen präsentiert und bedingt neben dem Auslösen einer Antikörperantwort auch die Induktion der zellulären Immunantwort, wie bspw. durch CD8+-T-Zellen [44]. Ein weiterer Applikationsweg ist die Verwendung einer sog. Gen-Kanone (Gene Gun*),* hierbei erfolgt der Transfer von Mikropartikeln, die an ihrer Oberfläche mit DNA beschichtet sind. Ein weiteres Verfahren basiert auf der Anwendung eines hohen Drucks (Biojector) zum Transfer der DNA in tiefere Hautschichten/Muskelgewebe. Mikronadeln und Elektroporation sind weitere Applikationsformen [45, 46].

Um eine starke Expression der für das Antigen codierenden Sequenz zu erzielen, erfolgt die Expression des Antigens häufig unter der Kontrolle des CMV-Promoters, eines starken, nicht gewebespezifischen Promoters. Als Vektoren für DNA-Impfstoffe dienen in der Regel Plasmide oder sog. Minicircle-DNAs. Plasmide sind ausgehend von Bakterienkulturen leicht in großen Mengen herstellbar und isolierbar. Wichtig ist, dass Lipopolysaccharid-(LPS-)Reste abgetrennt werden. Dies erfolgt durch eine Kombination chromatografischer Verfahren wie Ionentauscher und hydrophobe Interaktionschromatografie. Abschließend erfolgt eine Sterilfiltration der isolierten DNA [47].

Minicircle-DNA wird ebenfalls aus *E. coli* gewonnen. Hierbei wird das Antigen-codierende Plasmid mit einem Vektor, der für eine Rekombinase codiert, co-transfiziert. Die für die Plasmidreplikation erforderlichen Sequenzanteile auf dem Antigen-codierenden Plasmid sind von rekombinationsfähigen Sequenzen flankiert. Die co-exprimierte Rekombinase vermittelt nun eine homologe Rekombination, sodass 2 zirkuläre Moleküle entstehen: zum einen die Minicircle-DNA und zum anderen ein Miniplasmid, das die für die Replikation in *E. coli* erforderlichen Sequenzen und Restriktionsschnittstellen enthält. Durch entsprechende Restriktionsenzyme kann dieses Plasmid linearisiert und somit effizient abgetrennt werden [48].

DNA-Impfstoffe sind unadjuvantiert. Allerdings kann durch das Einfügen CG-reicher Sequenzen ein Adjuvanseffekt erzielt werden, indem TLR9 stimuliert wird. DNA verfügt jedoch per se über eine Adjuvanswirkung, vermittelt durch den „TBK1-STING-Pathway“, was zu einer Induktion von Typ-1-Interferonen führt [49].

In der Veterinärmedizin findet bereits eine Reihe von DNA-Impfstoffen Anwendung, so bspw. in Form eines Impfstoffs gegen das West-Nil-Virus (WNV) bei Pferden, der aber nicht mehr angewendet wird, und in der Fischzucht als Impfstoff gegen IHNV (infektiöses hämatopoetisches Nekrose-Virus; [50]).

In Indien wurde im Jahr 2021 ein DNA-Impfstoff gegen SARS-CoV‑2 zugelassen. Dieser Impfstoff (ZyCoV-D) ist Plasmid-basiert und codiert für das Spike-Protein von SARS-CoV‑2. Klinische Studien zeigten eine Effektivität von 67 % hinsichtlich der Verhinderung symptomatischer Erkrankung an Covid-19 [51].

Ein Vorteil von DNA-Impfstoffen besteht in ihrer leichten Herstellbarkeit. Da die Expression der codierenden Sequenz im Zielgewebe erfolgt, ist von einer weitgehend korrekten Faltung und posttranslationalen Modifikation des Antigens auszugehen. Im Unterschied zu mRNA ist DNA deutlich stabiler und erfordert eine deutlich weniger komplexe Transport- und Lagerungslogistik [46].

Risiken, die in der Anwendung DNA-basierter Impfstoffe gesehen werden, sind u. a. die Induktion von Autoimmunerkrankungen, ausgelöst durch die Bildung DNA-spezifischer Antikörper. Klinische Studien haben jedoch keine Hinweise auf das verstärkte Auftreten von Autoimmunerkrankungen im Zusammenhang mit der Anwendung von DNA-Impfstoffen ergeben [46].

Ein weiterer Aspekt ist das Risiko einer möglichen Integration der DNA-Vakzine in das Wirtsgenom und somit die Bildung von Mutationen (Deletionen, Frameshift-Mutationen, deregulierte Fusionsproteine etc.) wie auch, bei Insertion in regulatorische Elemente, eine deregulierte Genexpression. Ein weiteres Risiko besteht in einer persistierenden Expression des Antigens und damit der möglichen Ausbildung von Immuntoleranz.

Daten aus der veterinärmedizinischen Anwendung deuten jedoch darauf hin, dass Integrationsereignisse seltener sind als natürlicherweise vorkommende Mutationen. Dennoch erfordert diese Fragestellung weitere Untersuchungen, da sie von entscheidender Bedeutung für die Bewertung des Nutzen-Risiko-Verhältnisses ist.

## Antigen-beladene Nanopartikel zur Induktion einer breiten Immunantwort

Eine weitere Plattformtechnologie basiert auf der Verwendung von Nanopartikeln als Antigenträger. Dies umfasst eine sehr heterogene Gruppe. Anorganische oder chemisch-synthetisch hergestellte Polymere, Lipidvesikel, selbstaggregierende Proteine oder virale Strukturproteine, die Partikel bilden, sogenannte VLPs (Virus-Like Particles), können in Form von Nanopartikeln als Antigenträger dienen. Ziel dabei ist es, durch die hoch geordnete Struktur der Antigene auf der Oberfläche dieser Antigen-Carrier eine besonders robuste Induktion der Immunantwort, insbesondere der B‑Zellantwort, auslösen zu können [52].

An dieser Stelle sei exemplarisch auf Ferritin-basierte Nanopartikel eingegangen. Ferritine gehören zu einer Familie hoch konservierter supramolekularer Nanostrukturen, die eine wesentliche Rolle als Speicher für Fe^3+^-Ionen spielen. Bei der Verwendung von Ferritin-Nanopartikeln als Antigenträger wird häufig über eine Sortase-vermittelte Donor-Akzeptor-Kopplung die Beladung mit dem Antigen erzielt. Dabei kommt es zu einer hoch geordneten homogenen Beladung mit dem Antigen. Immunisierungen von Mäusen zeigten, dass es bedingt durch die Kopplung im Vergleich zum ungekoppelten Antigen zu einer deutlich stärkeren Immunantwort kommt. Weiterhin werden durch die Kopplung zusätzliche Epitope zugänglich, was zu einer Verbreiterung der Immunantwort führt [53, 54].

Neben der Kopplung von nur einem Antigen ist auch die Kopplung einer Antigenmischung möglich, was zur Entstehung von „Mixed Particles“ führt, also von Partikeln, die heterogen an ihrer Oberfläche beladen sind. Dies kann ein wesentlicher Faktor zur Verbreiterung der Immunantwort gegen verwandte Erreger sein. Da die verschiedenen Antigene so weit voneinander getrennt sind, kann es zu keinem „Clustering“ der B‑Zellrezeptoren kommen, da die für die verschiedenen Antigene spezifischen Zielstrukturen zu weit auseinander liegen. Vielmehr erfolgen unter diesen Bedingungen eine Bindung und somit Clustering der B‑Zellrezeptoren, die zwischen den verschiedenen Antigenen konservierte Epitope erkennen. Diese Induktion von Antikörpern, die zwischen den verschiedenen Isolaten/Antigenen konservierte Bereiche erkennen, führt zu einer Verbreiterung der Schutzwirkung, indem gezielt konservierte Bereiche erkannt werden [55].

Eine noch in der präklinischen Phase der Entwicklung befindliche Technologie basiert auf zellpermeablen Kapsiden des Hepatitis-B-Virus. Nanopartikel auf Basis von HBV-Kapsiden sind bereits seit Längerem bekannt. Das HBV-Kapsid ist strukturell genau charakterisiert. Es baut sich aus Dimeren des „Hepatitis-B-Core-Antigens“ (HBcAg) auf und besitzt sog. Spike Tips als prominentes Strukturelement. In der die Spike Tips formenden Region können leicht andere Peptide oder Proteine insertiert werden, ohne die Fähigkeit zum Aufbau von Kapsiden zu beeinträchtigen [56, 57]. Dies kann entweder kovalent durch Insertion erfolgen oder mittels eines Adapters, über den flexibel verschiedene Antigene an die Oberfläche des Kapsids gekoppelt werden. Ein solcher Adapter kann bspw. der „Strep-Tag“ sein, der wiederum die Beladung mit Fusionsproteinen aus einem monomeren Streptavidin und dem interessierenden Antigen ermöglicht, oder eine kovalente Kopplung des Antigens unter Verwendung des „Dog-Tag/Dog-Catcher“-Systems. In beiden Fällen führt dies wiederum zur Entstehung eines Antigenträgers, der an seiner Oberfläche in hoch geordneter Struktur das gewünschte Antigen trägt und somit die effiziente Induktion einer B‑Zellantwort ermöglicht [58, 59]. Eine weitere Modifikation beinhaltet die Fusion eines Zellpermeabilität vermittelnden Peptidmotivs über einen Linker an den Aminoterminus des Core*-*Proteins. Als Zellpermeabilität vermittelndes Peptidmotiv wird hierbei das sog. TLM-Peptid (Translocation Motive) verwendet, das aus einer amphiphilen Alpha-Helix besteht [60, 61]. Nach dem Zusammenbau zum Kapsid entsteht so ein Antigenträger, der an seiner Oberfläche das TLM-Peptid präsentiert und so die direkte Aufnahme des beladenen Antigen-Carriers in APCs ermöglicht und somit neben der B‑Zellantwort auch die robuste Induktion einer CTL-Antwort ermöglicht [58, 59]. Ein weiterer Vorteil dieser membranpermeablen Nanopartikel als Antigenträger ist die Möglichkeit zur nadelfreien Immunisierung mittels oraler oder nasaler Applikation. Die Membranpermeabilität erlaubt die Antigenaufnahme über die Schleimhaut des Nasen-Rachen-Raums und somit auch die Induktion einer lokalen Antikörperantwort, was insbesondere bei respiratorischen Erregern von hoher Relevanz ist.

## Fazit

Zusammenfassend zeigt sich, dass die SARS-CoV-2-Pandemie der Entwicklung neuer und innovativer Impfstoffkonzepte einen wesentlichen Schub gegeben hat. Dies gilt insbesondere für Plattformtechnologien, die im Falle von neuartigen Erregern eine rasche Adaptation der jeweiligen Plattform an den neuen Erreger ermöglichen und so den Zeitraum bis zur Verfügbarkeit eines Impfstoffs wesentlich verkürzen können. Dies ist bedingt durch die rasche Anpassung des Produktionsprozesses der jeweiligen Plattformtechnologie an den neuen Erreger und einen in vielen Schritten schneller handhabbaren Zulassungsprozess, da auf bereits vorhandene plattformspezifische Daten für die Zulassung zurückgegriffen werden kann.

Allerdings ist zu bedenken, dass die Immunogenität des jeweiligen Antigens ein spezifisches Charakteristikum ist, auf das nicht aus den sonstigen Daten, die für die jeweilige Plattform vorliegen, geschlossen werden kann. Zur Sicherstellung eines positiven Nutzen-Risiko-Verhältnisses, das eine entscheidende Grundlage für die Zulassung ist, sind also dennoch entsprechende Studien erforderlich.

### Infobox 1 Plattformtechnologien: Definition, Charakteristika und Typen

**Definition**

Als Plattformtechnologie werden in dieser Arbeit nachfolgend Ansätze bezeichnet, die auf einem stets identischen Grundgerüst basieren und sich nur hinsichtlich des Antigens unterscheiden. Dies können Vektorimpfstoffe sein, die auf modifizierten Viren basieren, wie auch mRNA- oder DNA-basierte Impfstoffe und Nanopartikel als Antigenträger, die flexibel mit dem jeweils relevanten Antigen beladen werden können.

**Charakteristika einer Impfstoffplattform**

Vielseitigkeit: Ein einziges Grundgerüst kann für verschiedene Impfstoffe verwendet werden.

Dies ermöglicht rasche Modifikation für neu auftretende Krankheiten, vereinfacht das Herstellungsverfahren und beschleunigt die Entwicklung sowie die Zulassung.

**Typen von Impfstoffplattformtechnologien**

- *Vektorimpfstoffe:* basieren auf modifizierten Viren als Träger für Antigene anderer Erreger.
- *mRNA-basierte Plattformen:* verwenden mRNA zur Codierung des gewünschten Antigens.
- *DNA-Impfstoffplattformen:* verwenden DNA zur Codierung des gewünschten Antigens, die transkribiert werden muss.
- *Protein-basierte Plattformen* (z. B. Virus-ähnliche Partikel).

## Abkürzungen

Ad26: Adenovirus-Typ 26
APCs: Antigen-präsentierende Zellen („antigen presenting cells“)
BAC: Bacterial Artificial Chromosome
CAR: Coxsackievirus and Adenovirus Receptor
CMV: Zytomegalievirus
CVA: Chorioallantois Vaccinia Virus Ankara
DsRNA: Doppelsträngige Ribonukleinsäure
EUA: Notfallzulassung (Emergency Use Authorization)
HEK293: Humane embryonale Nieren-293-Zellen (Human Embryonic Kidney)
HIV: Humanes Immundefizienz-Virus
IHNV: Infektiöses hämatopoetisches Nekrose-Virus
LDL: Low Density Lipoprotein
LGP2: Laboratory of Genetics and Physiology 2 (DHX58)
LINE‑1: Long Interspersed Nuclear Element
LNP: Lipidnanopartikel
MDA‑5: Melanoma Differentiation-Associated Protein 5
MVA: Modifiziertes Vacciniavirus Ankara
PEG: Polyethylenglycol
PRRs: Pattern Recognition Receptors
RIG‑I: Retinoic Acid-inducible Gene I
RSV: Respiratorisches Synzytial-Virus
SaRNA: Selbstamplifizierende Ribonukleinsäure
SARS-CoV‑2: Severe Acute RespiratorySyndrome Coronavirus Type 2
SsRNA: Einzelsträngige Ribonukleinsäure (Single-stranded RNA)
STING: Stimulator of Interferon Genes
TANK: TRAF Family Member-associated NF-kappa‑B Activator
TBK1: TANK-Binding Kinase 1
TLR: Toll-Like Receptor
UTR: Untranslatierte Region
VSV: Vesikuläres Stomatitis-Virus
WNV: West-Nil-Virus

## Footnotes

1 „Rolling-Review“: Bearbeitung einzelner Datenpakete bevor die kompletten Datenpakete zu den verschiedenen Aspekten der Zulassung wie Beschaffenheit des Impfstoffs, Sicherheit, präklinische Daten und Wirksamkeit vorliegen.
